# High-speed photoacoustic laser streaming and on-demand fluid mixing in a microfluidic chip with carbon nanotube-based elastomers

**DOI:** 10.1016/j.pacs.2026.100869

**Published:** 2026-08-09

**Authors:** Daxin Jiang, Peng Yu, Qichao Yang, Yizheng Wang, Chunhui Xu, Zhuochen Duan, Yiqian Zhang, Feng Lin, Jiming Bao, Chong Wang

**Affiliations:** Yunnan Key Laboratory for Micro/Nano Materials & Technology, School of Materials and Energy, Yunnan University, Kunming 650500, China; National Center for International Research on Photoelectric and Energy Materials, School of Materials and Energy, Yunnan University, Kunming 650500, China; Institute of Fundamental and Frontier Sciences, University of Electronic Science and Technology of China, Chengdu 611731, China; Yunnan Key Laboratory for Micro/Nano Materials & Technology, School of Materials and Energy, Yunnan University, Kunming 650500, China; National Center for International Research on Photoelectric and Energy Materials, School of Materials and Energy, Yunnan University, Kunming 650500, China; Yunnan Key Laboratory for Micro/Nano Materials & Technology, School of Materials and Energy, Yunnan University, Kunming 650500, China; Yunnan Key Laboratory for Micro/Nano Materials & Technology, School of Materials and Energy, Yunnan University, Kunming 650500, China; Department of Electrical and Computer Engineering, Texas Center for Superconductivity (TcSUH), University of Houston, Houston, TX 77204, USA; c National Center for International Research on Photoelectric and Energy Materials, School of Materials and Energy, Yunnan University, Kunming 650500, China; Department of Electrical and Computer Engineering, Texas Center for Superconductivity (TcSUH), University of Houston, Houston, TX 77204, USA; Yunnan Key Laboratory for Micro/Nano Materials & Technology, School of Materials and Energy, Yunnan University, Kunming 650500, China; National Center for International Research on Photoelectric and Energy Materials, School of Materials and Energy, Yunnan University, Kunming 650500, China

**Keywords:** Photoacoustic laser streaming, Microfluidic chip, Carbon nanotubes, Photoacoustic elastomer, Microfluidic mixer

## Abstract

Carbon nanotube (CNT)-based elastomers are renowned for generating high acoustic pressures via the photoacoustic effect, while little photoacoustic laser streaming (PALS) has been observed. In this work, we overcome this challenge by identifying laser spot diameter and repetition rate as two most important parameters governing PALS with CNT–polydimethylsiloxane (CNT–PDMS) composite elastomers. When a nanosecond laser with the repetition rate of 2000 Hz was focused to a 200 µm spot, we achieved a record-high PALS velocity of 28.1 cm/s. This soft, low-cost photoacoustic elastomer can mix two fluids within one minute in a milliliter-scale container, and it allows on-demand mixing of two laminar flows simply by switching on the laser without the need for microelectromechanical structures integrated into microfluidic channels. The high-speed PALS and controllable microfluidic mixing, established CNT composite elastomer as an excellent PALS medium and opens up new possibilities for practical photoacoustic microfluidics.

## 1 Introduction

Mixing of liquids is a fundamental operation in microfluidic chips [1], [2], microreactors [3], [4], and biomedical therapies [5], [6], [7], [8]. In microfluidic systems, liquids typically exhibit laminar flow within microchannels, resulting in immiscible flow and limited mixing efficiency [9], [10]. Micromixers are generally categorized into two types: passive and active. Passive micromixers such as hydrodynamic flow-focusing, toroidal, chaotic and baffled mixers, rely on channel microstructures to induce vortices or chaotic advection for enhanced mixing [11], [12], [13], [14], [15], [16], [17], [18]. In contrast, active micromixers can be switched on and off and utilize external energy inputs to drive fluid motion through mechanisms such as acoustic streaming, dielectrophoresis, and magnetohydrodynamic effects [10], [19]. Among these, piezoelectrically driven acoustic streaming is particularly attractive because it does not require specific electrical or magnetic properties of the fluid and is broadly applicable. However, because the streaming originates from surface piezoelectric actuation, such systems often involve complex fabrication processes, require electrical isolation from the fluid, and are not readily compatible with common microfluidic materials such as glass or soft polymers [19]. Even in well-established bubble-based acoustic mixing, where a trapped gas bubble inside a microfluidic channel is excited by a piezoelectric transducer outside the channel to generate streaming, practical challenges remain. These systems require the fabrication of microcavities within fluidic channels, and maintaining stable bubbles without dissolution or washout under continuous flow remains difficult [19], [20], [21].

Strong ultrasound waves have been frequently generated from carbon nanotube (CNT)-based elastomers through the photoacoustic effect for applications in imaging [22], surgery [6], printing [23], and delivery [23], [24]. However, despite producing acoustic pressures on the order of tens of megapascals (MPa) [25], [26], [27], [28], [29], [30], little to no fluid streaming has been observed [31], [32], [33], [34], [35]. A transient high-speed jet was previously produced under an acoustic pressure of ∼80 MPa using high laser pulse energy (80 mJ) and a large parabolic elastomer surface [6], [22], [23], [24], [36], [37], but this jet formed only at the focal point inside the fluid, with no measurable fluid motion near the elastomer surface [24], [31], [32], [33], [34], [35], [37]. Moreover, the high laser energy generated large bubbles on the elastomer surface, which can severely hinder practical applications [24], [37]. Recently, photoacoustic driving of microparticles and ink mixing were demonstrated by exciting a flat CNT-based self-healing elastomer with a strong laser beam (∼35 mJ/pulse). Despite producing acoustic pressures exceeding 25 MPa, the resulting flow velocity remained very low (∼1.5 mm/s) [25], [27]. In contrast, high-speed photoacoustic laser streaming (PALS) has been achieved at much lower acoustic pressures (∼0.2 MPa) from gold-implanted quartz plates or metallic surfaces [32], [34]. This striking contrast raises a fundamental question regarding the apparent discrepancy between high acoustic pressure and weak acoustic streaming.

In this work, we fabricated photoacoustic composite materials composed of CNTs and polydimethylsiloxane (PDMS), similar to typical elastomers, to identify the factors that have inhibited streaming generation in previous photoacoustic studies and to elucidate the operating principles of PALS in such composites. We demonstrate that the laser focus size and repetition rate are critical parameters governing PALS. Consequently, a high fluid velocity of 28.1 cm/s was achieved using a laser pulse energy of only 0.22 mJ. Rapid liquid mixing in a cuvette was further demonstrated as an example of macroscopic mixing. Moreover, efficient mixing of laminar flow was realized by simply directing a laser beam onto a CNT–PDMS film attached to the window of a microchannel, eliminating the need for complex microelectromechanical structures. The mixing function of this photoacoustic micromixer can be flexibly controlled by switching the incident laser on and off. This work reveals the key factors governing high-speed, continuous photoacoustic streaming and demonstrates its potential as a microfluidic pump and as a versatile macro–micro mixing platform for practical microfluidic applications.

## 2 Materials and methods

**Fig. 1:**
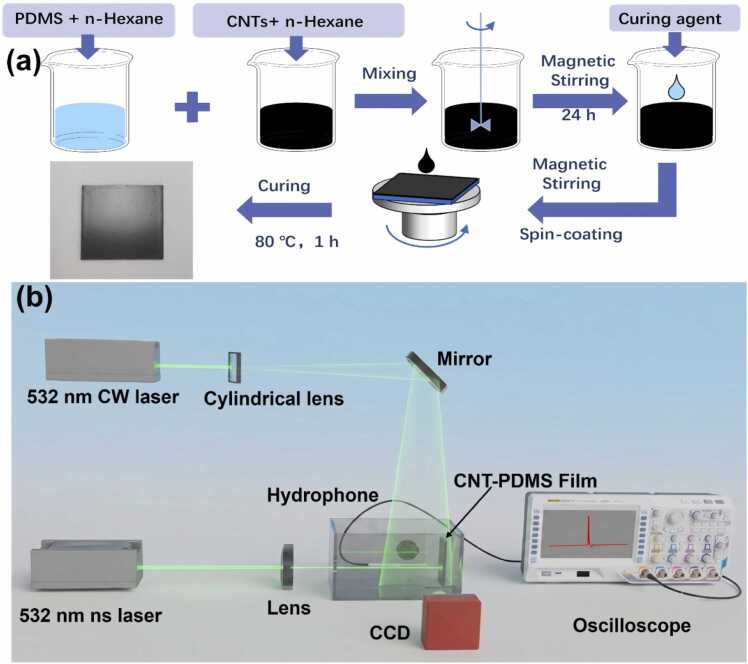
Preparation of the photoacoustic elastomer and schematic of photoacoustic laser streaming. (a) Preparation process of the CNT–PDMS elastomer film. (b) Schematic illustration of PALS using the CNT–PDMS elastomer film.

The CNT–PDMS composite film was prepared as a photoacoustic transducer elastomer following a procedure similar to previously reported elastomer-based methods, which involves spin-coating the CNT-PDMS dispersion onto a substrate [6], [22], [25], [38]. As shown in Fig. 1a, CNTs were dispersed in a mixed solvent of n-hexane and PDMS. After magnetic stirring, the CNT–PDMS dispersion was spin-coated onto a glass substrate and cured in an oven at 80 °C for 1 h to form a solid composite film. The CNT concentration and spin-coating speed were carefully adjusted to control film thickness (Fig. S1) and optical absorption. A CNT concentration of 8 wt% with optical penetration depth of ∼3.7 μm was selected for this study. Detailed preparation procedures are provided in the Materials Preparation section of the Supplementary Information (SI). As illustrated in Fig. 1b, the carbon-based composite elastomer was placed in a square glass container and excited by a focused 532 nm nanosecond laser (Coherent Matrix−532–8–100, pulse width: 25 ns, repetition rate: 1 Hz −10 kHz) incident on either the front or back side of the film. Fluorescent microspheres (Polystyrene, 5 µm in diameter; detailed information is shown in SI Section 1.2) were dispersed in water inside the container and illuminated from above by a continuous-wave (CW) 532 nm laser that passed through a cylindrical lens [31], [32], [33], [34], [35], [39]. Unless otherwise specified, the photoacoustic signal was detected using a hydrophone (Müller-Platte Needle Probe 100–100–1, diameter 1 mm) positioned 800 μm in front of the composite elastomer, and recorded with an oscilloscope (LeCroy WP7200A). The flow field was visualized with a camera (HY−800B), and a 532 nm notch filter was used to block the excitation laser. Flow velocity was determined using particle streak velocimetry (PSV), a technique widely used in the field and employed in our previous work [31], [32], [33], [34], [35], [40], [41], [42]. PSV calculates fluid velocity from the trajectory lengths of fluorescent microspheres during the camera exposure time under continuous-wave (CW) laser illumination. Steady-state velocity measurements in this study were conducted approximately 5 s after laser irradiation.

## 3 Results and discussion

### 3.1 PALS mechanism analysis of CNT-PDMS elastomer

**Fig. 2:**
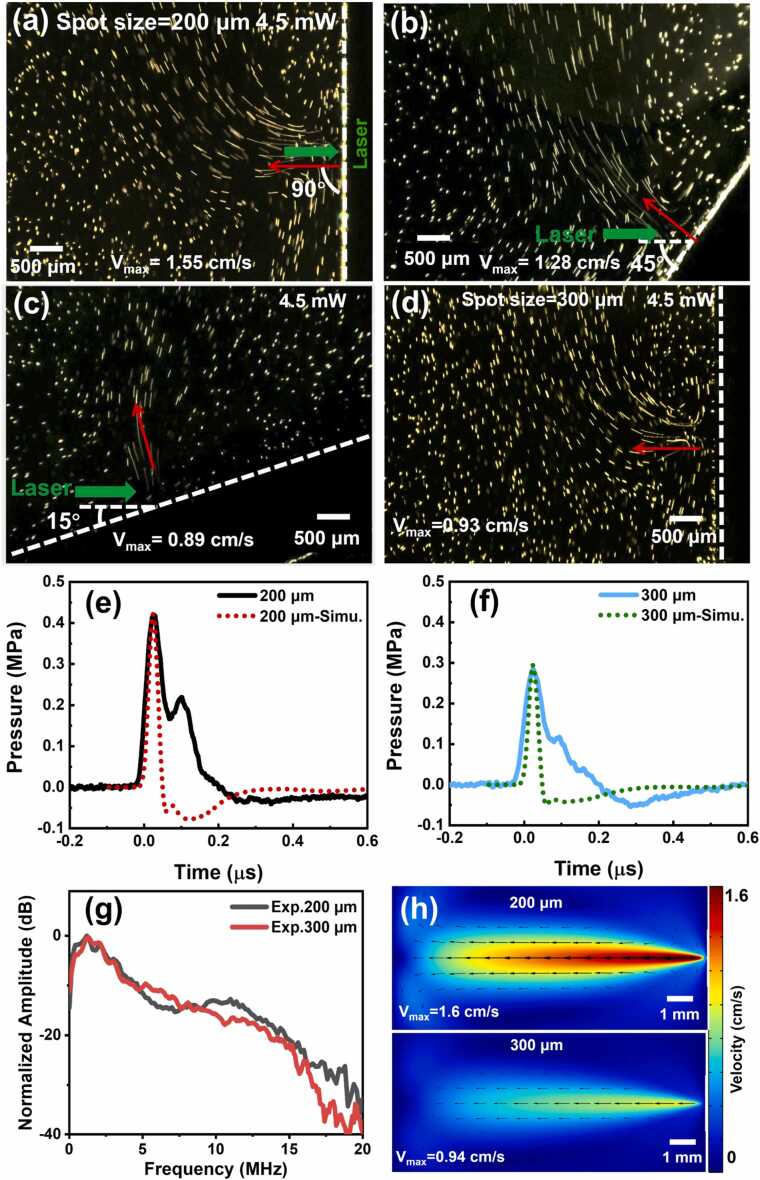
Mechanism analysis of PALS from CNT-PDMS film with tilt incident laser, low photoacoustic pressure, and different spot sizes. (a-c) Snapshots of jets generated at laser incidence angles of 90°, 45° and 15° relative to the substrate. The red arrows indicate the flow direction. The spot diameter is 200 μm and laser power is 4.5 mW. (d) Snapshots of PALS with laser spot diameter of 300 μm. The laser power is 4.5 mW. The white dashed line indicates the interface between water and the composite film. (e-f) Experimental and simulated photoacoustic pressure under laser illumination with (e) 200 μm and (f) 300 μm spot diameter. The laser power is 4.5 mW. (g) Fast Fourier Transform (FFT) spectra of the measured ultrasonic signals with 200 μm and 300 μm spot diameters. (h) Simulated PALS under laser illumination with 200 μm and 300 μm spot diameter. The film thickness is 10 μm.

PALS from the CNT–PDMS elastomer was first tested using a focused laser spot with a diameter of ∼200 µm, similar to the experimental parameters previously used for gold-implanted plates and metallic surfaces [31], [32], [33], [34], [35]. With a laser power of 4.5 mW and a repetition rate of 100 Hz, a jet with a velocity of ∼1.55 cm/s was generated, as shown in Fig. 2a. In Figs. 2a-2c, the jet remained perpendicular to the elastomer surface regardless of the laser incidence angle (90°, 45°, and 15° relative to the substrate), which is consistent with previous observations of PALS [32]. In contrast, the flow velocity decreased to only 0.93 cm/s with 4.5 mW laser illumination when the laser spot diameter was increased from 200 µm to 300 µm in Fig. 2d. In order to elucidate the driving mechanism and the influence of spot size, the corresponding photoacoustic pressures were measured and analyzed (Figs. 2e and 2f). The peak acoustic pressure generated by the 200 µm laser was ∼0.41 MPa (Fig. 2e), which is much stronger than the signal produced by the 300 µm spot (Fig. 2f) at the same 4.5 mW laser power. This amplitude is comparable to values reported in our earlier studies using Au-implanted quartz or metallic surfaces [32], [34]. For both spot sizes, the sharp positive pressure peaks in Figs. 2e and 2f reflect the rapid thermal expansion of the elastomer film under pulsed laser excitation, while the absence of a corresponding negative pressure pulse arises from the relatively slow cooling of the elastomer after the laser pulse. The weaker second peak at approximately 0.1 μs in Figs. 2e and 2f and Fig. S2 is attributed to detector ringing artifact [43]. To validate this PALS generation process and understand the influence of laser spot diameter on streaming, we performed simulations coupling thermoelastic with acoustic-fluid dynamics using COMSOL Multiphysics (Fig. S3; SI Section 2.2) [34]. The simulated transient acoustic pressures for both spot sizes agree well with the experimental measurements in Figs. 2e and 2f. Notably, the absence of the secondary peak in the simulation further confirms that this feature in the experimental data is merely a detector ringing artifact.

Beyond the time-domain waveform, the frequency spectrum of the acoustic signal is fundamentally related to its fluid-driving capability. Fast Fourier transform (FFT) analysis (Fig. 2g) reveals that the acoustic signals generated by the 200 µm and 300 µm laser spots exhibit a similar apparent center frequency of approximately 1.2 MHz. However, the 200 µm spot generates noticeably stronger high-frequency components. These enhanced high-frequency acoustic waves are crucial for efficient fluid actuation, as their rapid acoustic attenuation in water results in strong, localized momentum transfer [44]. Theoretically, the center frequency (*f*_*c*_) is related to the laser pulse width (*τ*_*l*_ ∼25 ns, full width at half maximum) and the stress relaxation time (*τ*_*r*_∼3.5 ns) by $f_{c}\approx \frac{1}{2(\tau_{l}+\tau_{r})}$, where *τ*_*r*_ corresponds to the time required for ultrasound to propagate across the optical penetration depth [26], [38], [45]. Because *τ*_*r*_ is significantly shorter than *τ*_*l*_, the frequency is dominated primarily by the *τ*_*l*_, yielding a theoretical center frequency of ∼17.5 MHz and a broadband acoustic signal [45]. In contrast, FFT analysis of the measured signal shows a much lower apparent center frequency around 1.2 MHz (expected to increase to ∼2 MHz after removing the detector ringing artifact). Despite this apparent discrepancy, the positive pressure pulse in the time domain exhibits a full width at half maximum (FWHM) of ∼30 ns, in good agreement with the theoretical effective pulse width ($\tau_{l}+\tau_{r}\text{=}28.5$ ns). The low apparent frequency in the FFT spectrum arises primarily from the broad negative-pressure tail associated with slow thermal relaxation after the passing of the laser pulse and from detector ringing within the transform window. In addition, the short-wavelength high-frequency components undergo significant attenuation during propagation through the PDMS and water.

To further investigate how the spot diameter affect the fluid-driving capability, the fluid simulations in Fig. 2h reveal a substantial decrease in the steady-state flow velocity when the laser spot diameter is expanded from 200 μm to 300 μm. Theoretically, this enlarged excitation area broadens the acoustic field and diminishes the spatial pressure gradient, which inherently weakens the localized Eckart-type driving force responsible for high-speed streaming. Furthermore, starting from a baseline temperature of 293.15 K, simulations of localized photothermal conversion at the water-nanocomposite interface (Fig. S4) show that the peak temperature reaches ∼320 K for the 200 μm spot, which is notably higher than the ∼303 K observed for the 300 μm spot. Importantly, although expanding the laser spot to 300 µm compromises some flow velocity, the correspondingly lower surface temperature reduces material degradation during prolonged irradiation (Fig. S5). Coupled with the convective cooling driven by the high-speed streaming, this effectively prevents thermal accumulation and enhances the long-term stable actuation of the film. The influence of laser spot diameter on streaming behavior can be understood from the fundamental principles of acoustic streaming, namely, fluid continuity and the pressure distribution within the acoustic field [31], [32], [34], [46].

These results differ markedly from prior studies employing carbon-based elastomeric transducers, where little to no streaming was observed despite acoustic pressures of tens of MPa [25], [26], [27], [28], [29], [30]. A closer examination of those experimental conditions reveals that their laser beams were unfocused, with spot diameters ranging from 2 to 8 mm, and their pulse repetition rate was limited to 20 Hz [26], [29], [30], [47], [48]. Such an unfocused spot generates a broad acoustic field lacking the steep spatial pressure gradients required to drive localized Eckart-type streaming [32], [34], [49], [50]. Furthermore, at a low repetition rate of 20 Hz, viscous drag completely dissipates the fluid's transient kinetic energy between consecutive pulses, preventing the temporal momentum accumulation necessary to sustain a steady-state flow [32], [51]. In contrast, in our focused-beam configuration, the laser spot diameter was smaller than the acoustic wavelength, allowing the generated acoustic wave to be approximated as a point source [34]. For instance, even when the laser power was further reduced to 3 mW (yielding a photoacoustic pressure of only 0.22 MPa), a jet velocity of 0.5 cm/s can still be observed in Fig. S6. The PALS velocity was higher than that of the CNT-PDMS film with 33.6 MPa photoacoustic pressure using unfocused laser beams [27]. In our observation of acoustic streaming, a small piezoelectric transducer under the same voltage generates acoustic streaming more readily than a larger one [31], [34]. We therefore propose that the laser spot diameter and pulse repetition rate, rather than the peak acoustic pressure alone, govern the strength of photoacoustic laser streaming.

### 3.2 PALS with different laser power and repetition rate

**Fig. 3:**
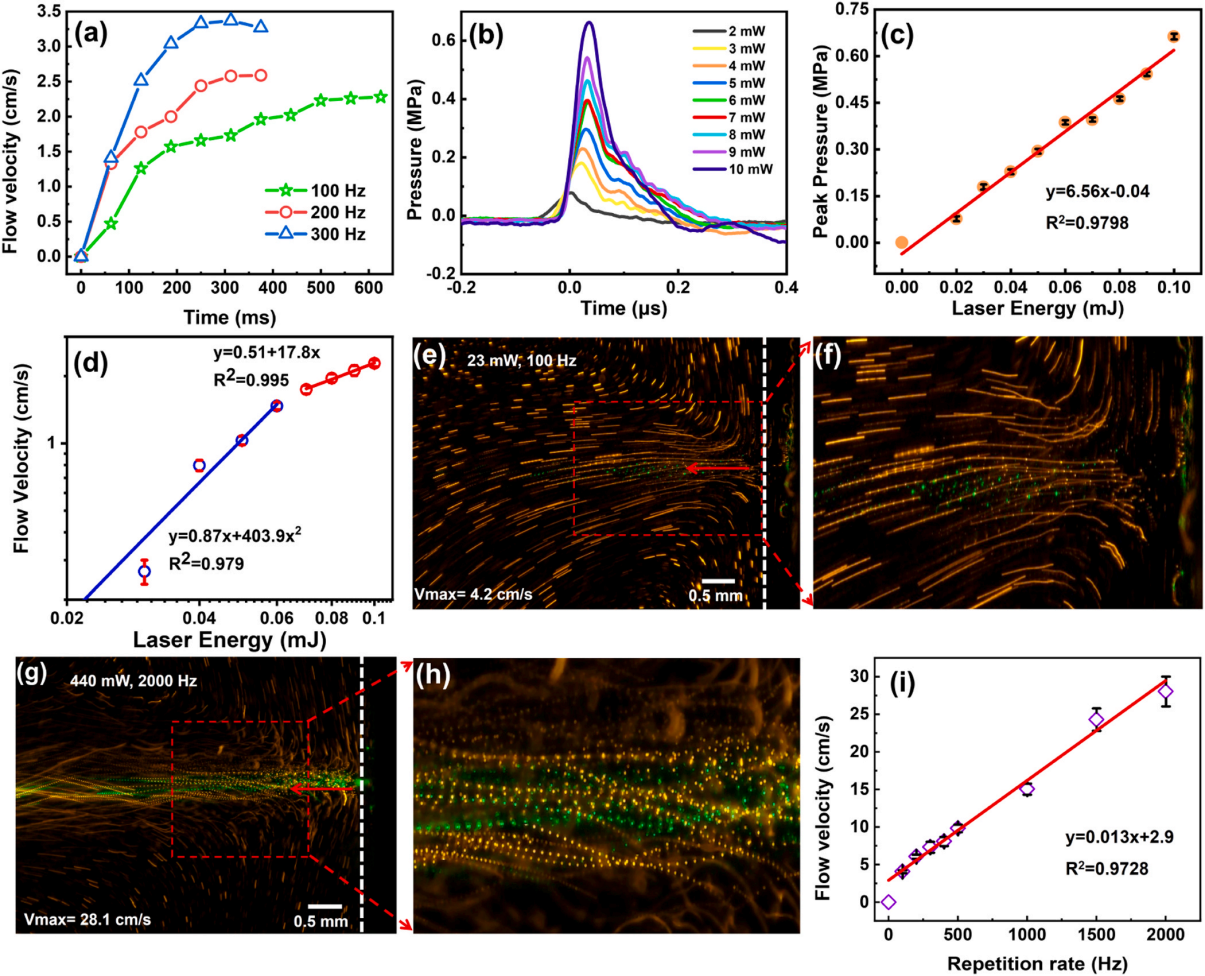
Speeds of PALS under increasing laser powers and repetition rates. (a) The acceleration of flow velocity under different laser repetition rates. The laser power is 10 mW and the spot diameter is 300 μm. The laser repetition rate is 100 Hz. (b) Photoacoustic pressures generated with increasing laser powers. (c) The linear dependence of photoacoustic peak pressure on laser pulse energy. (d) The relationship between flow velocity and laser energy. (e-h) Snapshots of PALS driven by lasers with repetition rates of (e-f) 100 Hz and (g-h) 2000 Hz. The red arrows indicate the flow direction. (i) Flow velocity as a function of the laser repetition rate. The spot diameter is about 300 μm. The film thickness is about 30 μm.

We emphasize that a jet can be generated by a single laser or pressure pulse, as demonstrated previously [32]. However, achieving a high steady-state flow velocity requires repeated laser excitation. Fig. 3a and Figs. S7–S9 illustrate the initial acceleration process: approximately 50 pulses are required for the flow to reach its terminal velocity, regardless of the laser repetition rate, corresponding to a transient time of 200–500 ms for repetition rates of 100–300 Hz [32], [35]. This rapid response, together with the excellent stability of the generated flow, demonstrates that the CNT–PDMS photoacoustic transducer is well suited for precise, on-demand microfluidic actuation and rapid fluid mixing [1], [2], [10]. Since a continuous stream is composed of individual transient jets generated by each laser pulse, increasing either the pulse energy or the repetition rate will increase the steady-state streaming velocity. As shown in Figs. 3b–3c, the peak acoustic pressure increases linearly with the laser pulse energy when the repetition rate is kept constant at 100 Hz. However, in Fig. 3d and Fig. S10, the relationship between the flow velocity and laser pulse energy exhibits a distinct two-regime behavior. At lower laser energies (<0.06 mJ), the streaming velocity scales quadratically with the laser energy. However, as the laser energy increases (>0.06 mJ), the dependence transitions to a linear regime. This scaling transition is consistent with the classical acoustic streaming research and can be explained by the balance between the acoustic driving force and hydrodynamic drag (*F*_*drag*_) under steady-state flow conditions [51]. At low laser energies (pressure), the generated flow is relatively slow and operates in a viscosity-dominated regime (Reynolds number ∼ 0.9, see SI Section 2.6). In this state, the viscous drag force scales linearly with velocity (*F*_*drag*_*∝v*) [52]. Since the acoustic driving force is proportional to the square of the acoustic pressure (*p* ^2^), balancing these two forces naturally results in a quadratic relationship between velocity and pressure (*v∝p*^*2*^). However, as the laser energy exceeds 0.06 mJ (reaching a velocity of 2.3 cm/s at 10 mW), the flow transitions into an inertia-dominated regime (Reynolds number ∼78 **≫** 1; see SI Section 2.6) where the drag force scales quadratically with velocity (*F*_*drag*_*∝v*^*2*^) [52]. Because the acoustic driving force scales with the square of the acoustic pressure (*p*^*2*^), equating these two forces yields a linear relationship between velocity and acoustic pressure (*v∝p*) [51], [52]. Fourier transform analysis of the photoacoustic pressure pulses in Fig. 3a reveals a stable center frequency and a broad acoustic bandwidth up to ∼13.3 MHz (−20 dB) within this energy range (Fig. S11), further confirming the linear relationship between pulse energy and pressure amplitude.

Fig. 3e–i and Fig. S12 show that the flow velocity further increases approximately linearly with the laser repetition rate (100–2000 Hz), reaching an impressive 28.1 cm/s at 2000 Hz (Supplementary Video 1)—over six times higher than previously reported PALS values [32], [33]. Based on experimental observation, a minimum repetition rate of ∼20 Hz is required to initiate continuous flow and the actual velocity drops sharply below 100 Hz. It should be noted that the local linear fit is only valid in continuous-flow regime, which can satisfy the applications demands of high-speed flow. These results indicate that the CNT–PDMS elastomer operates in a thermoelastic regime without structural degradation or collapse [25]. It is worth noting that such a linear dependence of flow velocity on pulse repetition rate was not observed in previous studies, as those systems were operated at a constant total acoustic power [50], [53]. We also found that repetition rates above ∼2 kHz led to permanent thermal damage of the composite film due to its limited heat dissipation. Therefore, a repetition rate near 2 kHz, combined with high total laser power, is preferred to achieve high flow velocity and efficient mixing.

### 3.3 PALS liquid mixing applications of CNT-PDMS elastomer

**Fig. 4:**
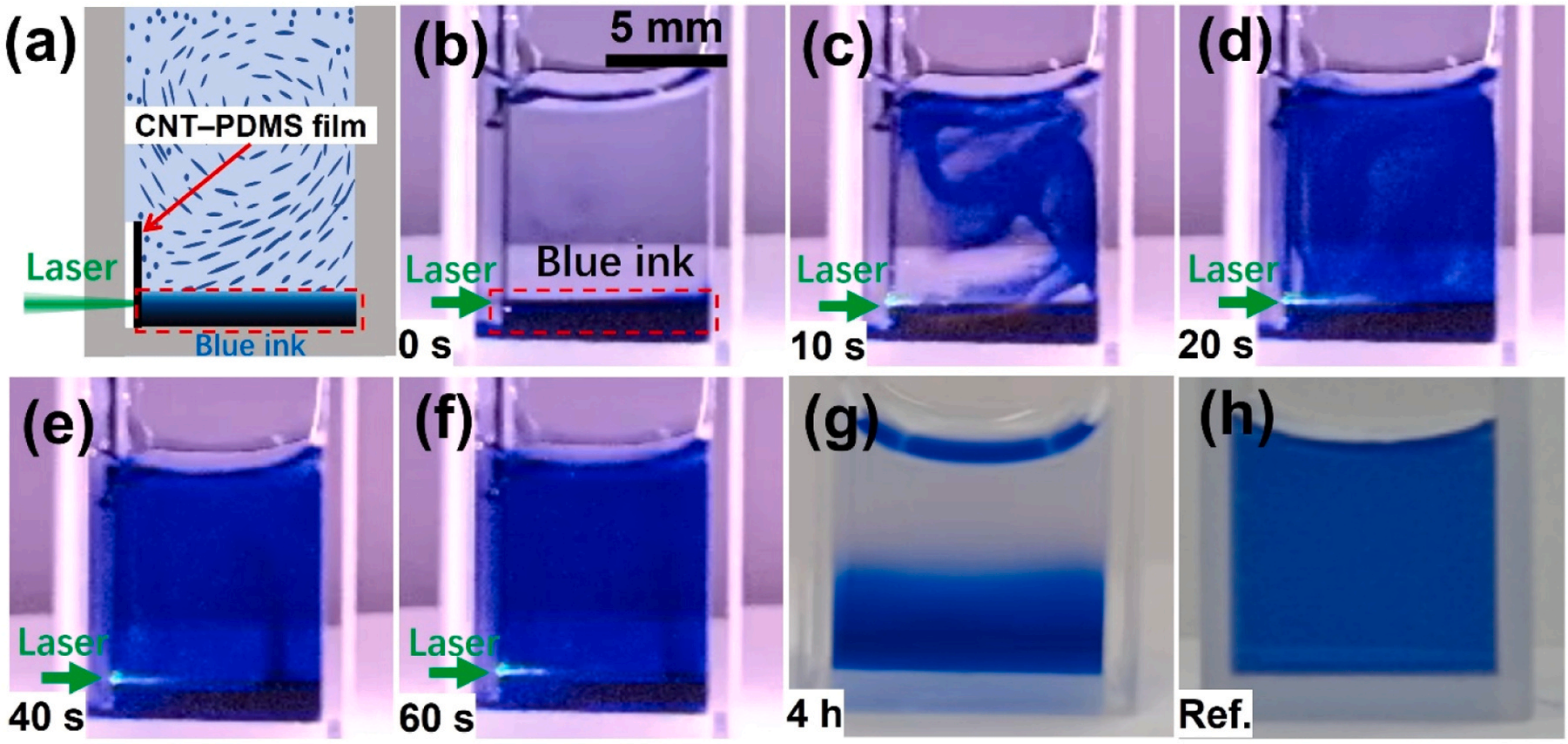
Mixing efficiency of two liquids using the CNT-PDMS photoacoustic transducer and free diffusion. (a) Schematic of liquid mixing in cuvette using CNT-PDMS transducer. The CNT-PDMS film was coated on a glass plate and vertically placed in the cuvette. (b-f) Snapshots of liquid mixing driven by CNT-PDMS PALS. A 532 nm notch filter was placed in front of the camera to block the excitation laser. (g-h) Snapshots of free-diffusion mixing after 4 h and 12 h. The laser power was 250 mW, and the repetition rate was 2000 Hz. The film thickness is 30 μm. The green arrow indicates the incident 532 nm nanosecond laser. The spot diameter is about 300 μm.

The high-speed PALS enables the CNT–PDMS composite to be applied in microfluidic systems. As a demonstration, we first performed the mixing of ink and water. A glass plate coated with a CNT–PDMS film was placed vertically in a cuvette containing 10 µL of ink at the bottom and 1 ml of water above it, as illustrated in the schematic of Fig. 4a. The elastomer film was attached to the inner side wall near the bottom of the cuvette and excited from the outside by a laser beam perpendicular to the back side of the film. As shown in Fig. 4b–g and Supplementary Video 3, once the laser was turned on, a jet was generated at the water–ink interface, initiating rapid mixing between the two liquids. After 60 s of irradiation, the solution became nearly uniform, comparable to the manually mixed reference sample (Fig. 4h). In contrast, without laser excitation, the water and ink remained clearly separated even after 4 h (Fig. 4g). The solution finally reached uniform mixing after 12 h free diffusion in Fig. 4h and Fig. S13.

**Fig. 5:**
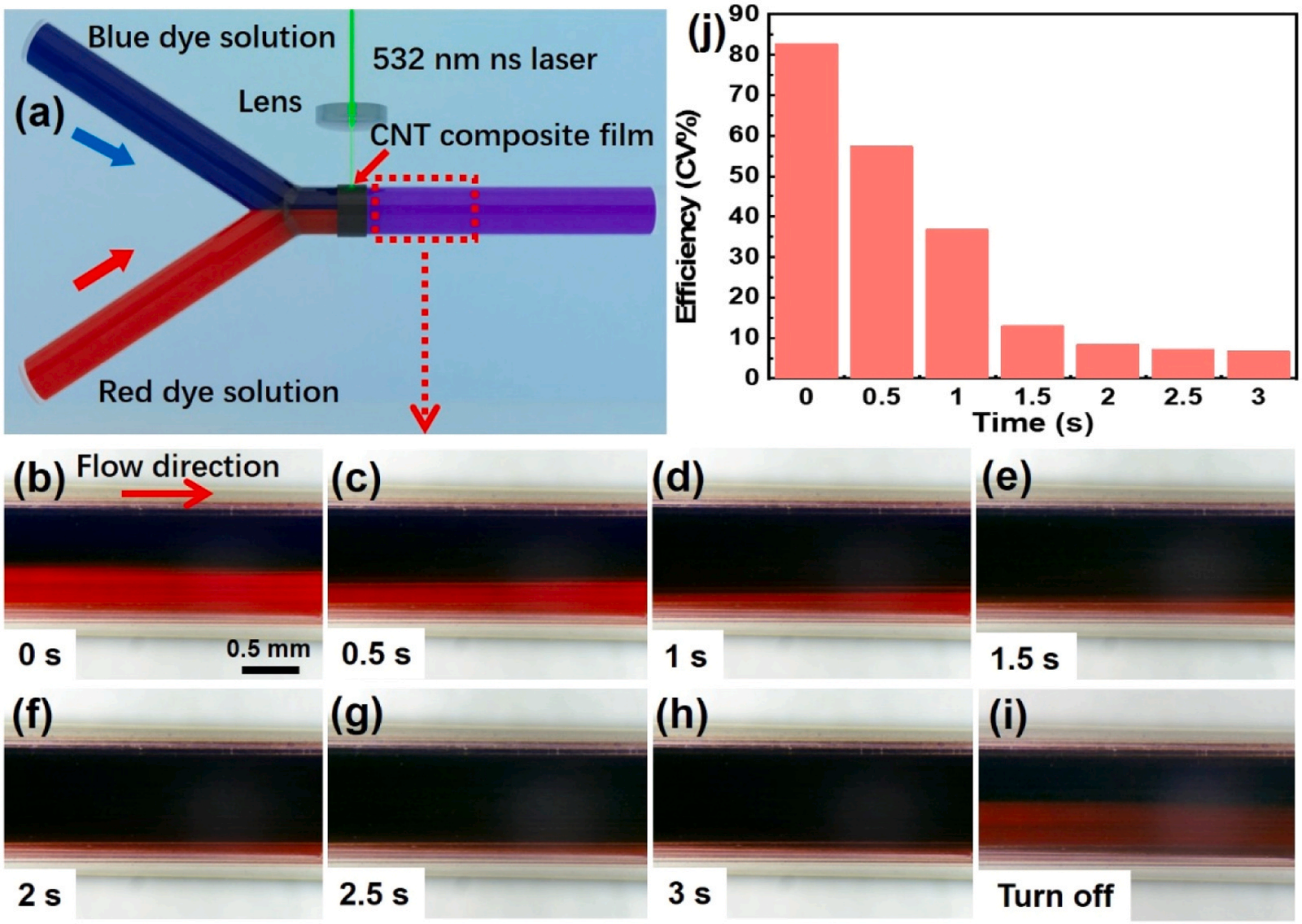
Photoacoustic CNT-PDMS micromixer. (a) Side-view schematic of the photoacoustic micromixer. The dashed box indicates the flow observation region. (b) Laminar flow of two dye solutions in the microchannel before laser excitation. (c-h) Time-evolution snapshots of mixing at 0.5 s (c), 1 s (d), 1.5 s (e), 2 s (f), 2.5 s (g), and 3 s (h). (i) Snapshots of the flow after the laser was turned off. (j) Mixing efficiency of blue and red dyes in the microchannel. The flow velocity was 0.2 mm/s, the laser power was 150 mW, and the repetition rate was 2000 Hz. The film thickness is 31 μm. The spot diameter is about 300 μm.

Since efficient fluid mixing is highly desirable in microcontainers and microchannels, the rapid mixing capability of the CNT–PDMS elastomer suggests its strong potential as a photoacoustic micromixer for microfluidic applications [9], [11], [12], [13], [14], [15], [16], [17], [18], [19], [54]. In the experiment of Fig. 5, the CNT–PDMS transducer film was integrated onto a microchannel as a photoacoustic micromixer. As illustrated in Fig. 5a, the transducer film was positioned to cover a small opening in a standard, pre-fabricated circular mixing channel (diameter: 500 μm). This configuration can also be readily implemented by placing a microfluidic chip onto a substrate coated with the transducer film, without requiring any modification to the channel itself. Fig. 5b shows the typical unmixed laminar flow of two inlet liquids in the microchannel, where mixing is inherently difficult without additional structural features. In contrast, in our setup, effective mixing was readily achieved by simply controlling the incident laser. When the laser irradiated the back side of the transducer film, the two liquids mixed homogeneously within approximately 3 s, as shown in Fig. 5c–h and Supplementary Video 4. Once the laser was turned off, the mixed flow quickly reverted to its original laminar state (Fig. 5i). To quantitatively analyze the mixing process, the coefficient of variation (CV %) was selected to describe the mixing efficiency and calculated using an imaging processing tool (ImageJ, Fiji) [31]. The CV% was calculated with the equation CV%= (*σ/μ*)× 100%, where *σ* represents the standard deviation and *μ* represents the mean of gray values [31]. The middle areas of the microchannel in Figs. 5b-5i were selected as the region of interest. Fig. 5j shows the temporal evolution of the mixing efficiency CV. The CV decreased rapidly from 82.5% to 12.7% within 1.5 s and reached a steady value of 6.6% after 3 s, confirming the high uniformity and effectiveness of photoacoustic mixing. It is worth noting that, compared to traditional acoustic streaming–based micromixing techniques such as surface acoustic waves [55] and bubble mixing [19], [20], [21], our method offers a distinct advantage in throughput. Our device maintains efficient mixing at a flow rate of 3240 μL/min (54 μL/s), representing an approximately two-orders-of-magnitude increase compared to conventional acoustic bubble mixing (16 μL/min) [37]. Fundamentally, the mixing time is governed by the fluid's residence time within the active zone, which is dictated by the competition between the transverse photoacoustic streaming and the axial flow rate. As detailed in Fig. S14, decreasing the baseline flow rate to 1.2 μL/s further shortens the required mixing time to 1.5 s, whereas an excessively high flow rate (140 μL/s) critically limits the residence time and prevents uniform mixing. This performance suggests that our design is particularly well suited for applications requiring both rapid mixing and high-throughput processing.

## 4 Conclusion

In summary, we resolved the long-standing paradox of weak PALS despite high acoustic pressures in carbon-based elastomers and established CNT–PDMS elastomers as a flexible, low-cost platform for PALS. By employing a focused laser spot and a high repetition rate, we achieved high-speed laser streaming with a record flow velocity of 28.1 cm/s using only 0.22 mJ pulse energy at 2 kHz. Based on this versatile transducer film, we demonstrated both a CNT–PDMS microfluidic pump and rapid macroscopic mixing of milliliter-scale liquids within 60 s. Furthermore, by simply attaching the soft elastomer film onto microchannels, we realized an efficient photoacoustic micromixer capable of homogenizing laminar flows within seconds. This photoacoustic film eliminates the need for costly microfabrication associated with conventional passive and active micromixers, while maintaining excellent portability and compatibility with microfluidic systems. The demonstrated high-speed PALS not only positions the CNT–PDMS elastomer as an outstanding medium for laser-driven flow generation but also opens new avenues for integrating photoacoustic actuation into practical microfluidic technologies.

## CRediT authorship contribution statement

**Yiqian Zhang:** Methodology, Investigation. **Feng Lin:** Writing – review & editing, Writing – original draft, Visualization, Validation, Supervision, Software, Resources, Project administration, Methodology, Formal analysis, Conceptualization. **Jiming Bao:** Writing – review & editing, Supervision, Project administration, Methodology, Conceptualization. **Chong Wang:** Writing – review & editing, Writing – original draft, Supervision, Resources, Project administration, Funding acquisition, Formal analysis. **Daxin Jiang:** Writing – original draft, Methodology, Investigation, Formal analysis, Data curation, Conceptualization. **Peng Yu:** Writing – original draft, Methodology, Investigation, Formal analysis. **Qichao Yang:** Methodology, Investigation, Formal analysis. **Yizheng Wang:** Methodology, Investigation, Formal analysis. **Chunhui Xu:** Methodology, Investigation. **Zhuochen Duan:** Methodology, Investigation.

## Declaration of Competing Interest

The authors declare that they have no known competing financial interests or personal relationships that could have appeared to influence the work reported in this paper.

## Data Availability

Data will be made available on request.
