# Supplementary material for: High-speed photoacoustic laser streaming and on-demand fluid mixing in a microfluidic chip with carbon nanotube-based elastomers
Source: Photoacoustics. 2026 Aug 9;51:100869. doi: 10.1016/j.pacs.2026.100869 (PMC13482216; doi:10.1016/j.pacs.2026.100869)
Supplement: Supplementary file 1 [file mmc1.docx]

**Supplementary Information**

**High-Speed Photoacoustic Laser Streaming and On-Demand Fluid Mixing in a Microfluidic Chip with Carbon Nanotube-Based Elastomers**

Daxin Jiang^1,3†^, Peng Yu^4†^, Qichao Yang^1,3^, Yizheng Wang^1,3^, Chunhui Xu^1,3^, Zhuochen Duan^1,3^, Yiqian Zhang^1^, Feng Lin^1,2,3^*, Jiming Bao^2^*, Chong Wang^1,3^*

1. Yunnan Key Laboratory for Micro/Nano Materials & Technology, School of Materials and Energy, Yunnan University, Kunming 650500, China
2. Department of Electrical and Computer Engineering, Texas Center for Superconductivity (TcSUH), University of Houston, Houston, Texas 77204, USA
3. National Center for International Research on Photoelectric and Energy Materials, School of Materials and Energy, Yunnan University, Kunming 650500, China
4. Institute of Fundamental and Frontier Sciences, University of Electronic Science and Technology of China, Chengdu 611731, China

**1. Materials and Methods**

**1.1. CNT elastomer film preparation**

The carbon nanotubes (CNTs) (tube diameter: 10 nm and tube length: 15 μm) was purchased from Suiheng Technology, Shenzhen, China and the PDMS is Sylgard 184. During the preparation of films, the quartz substrate must be ultrasonically cleaned and rinsed with acetone, ethanol, and deionized water to thoroughly remove grease and dust. After drying, it is treated with ozone for 30 minutes to enhance the hydrophilicity of the substrate.

Then, 1 g of polymer prepolymer is first weighed into a sample vial, and 10 mL of n-hexane is added until the polymer is completely dissolved. Next, the required mass of the CNT is calculated based on the total mass of the polymer and the curing agent according to a predetermined mass ratio. Based on this calculation, 95.7 mg (8 wt%) of CNT is weighed into a sample vial, and n-hexane is added to achieve a dispersion concentration of 15 mg/mL. Subsequently, the carbon nanomaterial dispersion is placed in a beaker within a water bath and subjected to probe sonication for 30 minutes, during which the temperature must be maintained below 30°C. The polymer dispersion undergoes the exact same ultrasonic treatment for 9 minutes. The ultrasonically dispersed polymer solution and the CNT solution are then mixed uniformly. A magnetic stir bar is added to the mixture, which is placed on a magnetic stirrer and stirred for 24 hours. This allows the n-hexane to evaporate while ensuring the CNT are evenly dispersed throughout the polymer. Following this, a curing agent is added to the mixture at a 1:10 ratio of curing agent to PDMS prepolymer. Once the polymer prepolymer and curing agent are thoroughly stirred, the mixture is transferred onto the quartz substrate for spin coating. Films of varying thicknesses are obtained by controlling the spin-coating time and speed. The spin-coated film is then subjected to a vacuum treatment for 30 minutes to remove any trapped air bubbles. Finally, the treated film is placed in a forced-air drying oven (blast drying oven) and cured at 80°C for 1 hour to yield the photoacoustic driving film.

The spin-coating time was kept constant at 60 s for all samples. Films of various thicknesses were prepared by adjusting the spin coating speed (6000 rpm to 9000 rpm). The relationship between the spin-coating speed and the resulting film thickness is illustrated in Figure S1.

**Fig. S1** Film thickness as a function of spin coating speed.

**1.2. Fluorescent microsphere**

Fluorescent microspheres (Polystyrene, emitting yellow light at 580 nm, 5 µm in diameter) were dispersed in water inside the container and illuminated from above by a continuous-wave (CW) 532 nm laser that passed through a cylindrical lens to track the flow. During experiment, only one drop of particle solution was used in a glass container to keep low particle concentration for clear tracking of flow^1^. The reason we choose 5 μm particle is to guarantee strong optically trackable signal because too small particles cannot emit or scatter enough fluorescence to be clearly identified and tracked. However, particles with too large size will experience high flow resistance and affect fluid dynamic tracking ability. We utilized fluorescent polystyrene microspheres with a density of *ρ_p_*=1050 kg/m³. The density of water at 20 ℃ is *ρ_f_*=998.2 kg/m³. The dynamic viscosity of water at 20 ℃ is *μ*=1.002×10^−3^ Pa·s and the maximum observed flow velocity of *v_max_*=0.281 m/s, the calculated Stokes number in the most critical^2^, high-speed region is indeed $\text{S}_{\text{t}}\text{=}\frac{\text{d}_{\text{p}}\text{|}\text{ρ}_{\text{p}}\text{-}\text{ρ}_{\text{f}}\text{|}\text{v}_{\text{max}}}{\text{μ}}$≈0.0726. Since this value strictly satisfies the condition *St*≪1, it confirms that the particle inertia is negligible, and they behave as highly accurate tracers for the fluid flow.

To address the concern regarding the influence of the acoustic field on the particles, we compared the acoustic radiation force (*F_rad_*) and the drag force (*F_drag_*)^3^. In experiment, the wavelength (*λ*) of acoustic wave with central frequency (*f*) of 1.2 MHz is *λ=u/f*=1.23 mm, where *u* (1480 m/s) is the sound velocity in water. For a spherical particle with diameter much smaller than the sound wavelength *λ*, the primary acoustic radiation force is *F_rad_*=$\text{-}\text{∇}\text{U}^{\text{rad}}$ and $\text{U}^{\text{rad}}\text{=}\frac{\text{4π}}{\text{3}}({\frac{d_{p}}{2})}^{3}\text{[}\text{f}_{\text{1}}\frac{\text{1}}{\text{2}}\text{κ}_{\text{0}}\text{<}\text{p}_{\text{in}}^{\text{2}}\text{>-}\text{f}_{\text{2}}\frac{\text{3}}{\text{4}}\text{ρ}_{\text{0}}\text{<}\text{v}_{\text{in}}^{\text{2}}\text{>]}$, where *d_p_* is the particle diameter, *κ_0_* is the compressibility, *ρ_0_* is the density of water, *f_1_* is the monopole coefficient, *f_2_* is the dipole coefficient, <$\text{p}_{\text{in}}^{\text{2}}$>is the mean square value of sound pressure, and <$\text{v}_{\text{in}}^{\text{2}}$> is the mean square value of acoustic particle velocity^4^. The compressibility of water *κ_0_=*$\frac{\text{1}}{\text{ρ}_{\text{0}}\text{c}_{\text{0}}^{\text{2}}}$ =4.44*×10^-10^ Pa^-1^*. The compressibility of polystyrene *κ_p_=*$\frac{\text{1}}{\text{ρ}_{\text{p}}\text{c}_{\text{p}}^{\text{2}}}$*=*$\frac{\text{1}}{\text{1050×}\text{2400}^{\text{2}}}$*=*1.653×10^-10^ Pa^-1^. The monopole coefficient *f_1_*=1-$\frac{\text{κ}_{\text{p}}}{\text{κ}_{\text{0}}}$=0.628. The dipole coefficient *f_2_=*$\frac{\text{2}\text{（}\text{ρ}_{\text{p}}\text{-}\text{ρ}_{\text{0}}\text{）}}{\text{2}\text{ρ}_{\text{p}}\text{+}\text{ρ}_{\text{0}}}$*=0.032.* The mean square value of sound pressure <$\text{p}_{\text{in}}^{\text{2}}$>=$\frac{P_{a}^{2}}{2}$=1.02×10^12^ Pa, where *P_a_* (1.43×10^6^ Pa) is the maximum acoustic pressure. The mean square value of acoustic particle velocity <$\text{v}_{\text{in}}^{\text{2}}$>=$\frac{\text{v}_{\text{a}}^{\text{2}}}{\text{2}}$=$\frac{\text{1}}{\text{2}}{\text{(}\frac{\text{P}_{\text{a}}}{\text{ρ}_{\text{0}}\text{c}_{\text{0}}}\text{)}}^{\text{2}}$=0.4544 (m/s)^2^. Therefore, we can obtain *U^rad^* as 8.59×10^-15^ J and *F_rad_*=$\text{-}\text{∇}\text{U}^{\text{rad}}$=$\frac{\text{U}^{\text{rad}}}{\text{Δx}}$=$\frac{\text{8.59×}\text{10}^{\text{-15}}}{\text{3.16}\text{×}\text{10}^{\text{-4}}}$=2.7×10^-11^ N when the laser spot size is 316 μm. In contrast, the fluid drag force is *F_drag_=3πμd_p_v*, where *μ* is the dynamic viscosity of water, *d_p_* is the particle diameter, and *v* is the fluid velocity^5^. Therefore, the *F_drag_=*3π*×*1.002×10^-3^ *Pa∙s×*5×10^-6^ *m×*0.281 *m/s=*1.33×10^-8^ *N*. Because we selected a relatively small micro-particle (5 µm), the volume-dependent acoustic radiation force scales down dramatically and becomes orders of magnitude smaller than the drag force (*F_drag_* ≫ *F_rad_*). Therefore, the motion of the 5 µm particles is entirely dominated by fluid flow rather than acoustic propulsion.

**1.3. Flow Velocity Measurement**

The velocity measurement method employs polystyrene microspheres with a diameter of 5 μm (emitting yellow light at 580 nm.) These microspheres are illuminated by a 532 nm continuous-wave laser shaped into a light sheet via a cylindrical lens. By adjusting the exposure time of the camera, trajectories left by the yellow microspheres during the exposure period can be captured in the field of view^6-8^. The microsphere velocity ($\text{v}_{\text{t}}$) which corresponds to the flow velocity, is calculated by dividing the trajectory length $\text{l}_{\text{t}}$ by the exposure time $\text{t}_{\text{e}}$:

$$\text{v}_{\text{t}}\text{=}\frac{\text{l}_{\text{t}}}{\text{t}_{\text{e}}}$$

However, when the flow velocity is too high to capture a complete trajectory within the camera’s field of view, an alternative approach is used to determine the flow velocity. Since the pulsed laser also operates at 532 nm, the microspheres are similarly illuminated by the pulsed laser. Each laser pulse produces a unique image of the microsphere, and over the camera’s exposure time, a series of such microsphere images is formed, corresponding to the repetition rate of the pulsed laser (as shown in Figs. 3e-h in the main text). The instantaneous velocity of the microsphere ($\text{v}_{\text{s}}$) which represents the instantaneous flow velocity, is calculated by multiplying the distance $\text{l}_{\text{s}}$ between two adjacent microsphere images by the pulse repetition rate *f* (Hz):

$$\text{v}_{\text{s}}\text{=}\text{l}_{\text{s}}\text{⋅}\text{f}$$

During a typical flow velocity measurement, we recorded videos with a duration exceeding 10 seconds. From these videos, we selected several individual frames at intervals (more than 10 frames). We measured the longest streak produced by the exposure of the polystyrene microspheres in each frame, using this measurement to represent the maximum flow velocity for that specific frame. The overall velocity of the flow field was then determined by calculating the average of these maximum velocity values. This identical methodology was applied to determine the flow field velocity in the high-speed actuation videos. Regarding the measurement range of the flow velocity, we defined the maximum flow velocity within a single frame by selecting the longest streak or the maximum particle spacing that originated near the acoustic source. The error bars presented in the main text represent the standard deviation of these frame-by-frame measurements.

**1.4. Laser Beam Diameter Measurement**

We use a USB-CCD Laser Camera (Sony 960H CCD Sensor) to measure the focused laser spot size by calculating the full width at half maximum (FWHM) of its intensity profile.

**1.5. Camera Information**

In our experiments, the flow field was recorded using a commercial CCD camera (Model: HY-800B, Hayear). The detailed imaging parameters have been carefully reviewed and are listed as follows:

Pixel Resolution: 3840 × 2160 (4K)

Spatial Resolution: 2 µm/pixel: 2 μm (H) × 2 μm (V), calibrated by a standard micrometer under the objective lens

Spectral Response: 400 nm~1000 nm

Signal-to-Noise Ratio: 39 dB

Exposure Time: 62.5 ms (corresponding to an effective frame rate of 16 frames per second)

Exposure Gain: 100%

Color Temperature: 4600 K

**2. Supplementary Results and Discussion**

**2.1. Photoacoustic Signal and Fast Fourier Transform (FFT) with Different Spot Size**

**Fig. S2** Photoacoustic pressure of CNT-PDMS film illuminated by laser with different spot size. (a) Photoacoustic pressure of CNT-PDMS film with laser spot size of 200 μm and 300 μm. The fluence is about 140 mJ/cm^2^ for two laser spot sizes. (b) Normalized photoacoustic pressure in (a). (c) The normalized frequency spectrum of pressure signal in (a).

The Fast Fourier Transform (FFT) spectrum of the ultrasonic signal in Fig. 2d is shown in Fig. 2f. The frequency spectrum of the generated photoacoustic pulse is intrinsically linked to the laser pulse width (*τ_l_*) and the stress relaxation time (*τ_r_*) as $\text{f}_{\text{c}}\text{≈}\frac{\text{1}}{\text{2(}\text{τ}_{\text{l}}\text{+}\text{τ}_{\text{r}}\text{)}}$, where *f_c_* is the central frequency^9^. The *τ_r_* is defined as the time it takes for an acoustic wave to traverse the optical heating zone: *τ_r_=δ/c* where *δ* is the optical penetration depth and *c* is the speed of sound in the medium. Furthermore, governed by the time-frequency inverse relationship, a shorter *τ_r_* produces a narrower acoustic pulse in the time domain, thereby intrinsically broadening the frequency bandwidth (*Δf*) of the corresponding photoacoustic spectrum. Based on our measurements, the laser penetration depth (*δ*) in the CNT-PDMS composite is 3.7 μm. Assuming a typical speed of sound for PDMS (*c*≈1050 m/s), the acoustic relaxation time is exceptionally short, approximately 3.5 ns. Because this relaxation time is much shorter than the excitation laser pulse width (~25 ns), the system is not in a state of stress confinement. Therefore, the acoustic wave leaves the optical heating volume while the laser is still depositing energy, causing the temporal profile (and the broad frequency spectrum) of the acoustic pulse to be dictated primarily by the 25 ns laser pulse width rather than the spatial scale of optical absorption.

**2.2. Simulation of Photoacoustic Pressure and Photoacoustic Laser Streaming**

The photoacoustic pressure, temperature distribution, and photoacoustic laser streaming of the CNT-PDMS elastomer were simulated with COMSOL Multiphysics. Fig. S5 shows the rotational symmetric geometry of the simulation model. The layer of the model on the bottom is the glass substrate, the thin layer on the glass substrate is the CNT-PDMS elastomer film, and the bulk on the top is the water. The CNT-PDMS elastomer film was illuminated by nanosecond laser from the back side. Heat transfer module, solid mechanics module, thermoviscous acoustics module, and laminar flow module were coupled in the simulation model. The incident nanosecond laser was defined as

$\frac{\frac{\text{p}}{\text{f}}\text{α×}\left( \text{2}\sqrt{\text{2ln2}} \right)^{\text{3}}}{{\text{σ}_{\text{xy}}}^{\text{2}}\text{σ}_{\text{t}}\text{(2π}\text{)}^{\text{1.5}}}\text{exp}\left( \text{-}\frac{\left( \text{t-}\text{t}_{\text{0}} \right)^{\text{2}}}{\text{2}\left( \frac{\text{σ}_{\text{t}}}{\text{2}\sqrt{\text{2ln2}}} \right)^{\text{2}}} \right)\text{∙exp(-}\frac{\text{r}^{\text{2}}}{\text{2}\left( \frac{\text{σ}_{\text{xy}}}{\text{2}\sqrt{\text{2ln2}}} \right)^{\text{2}}}\text{)∙exp(-α(}\text{th}\text{-z))}$,

where *p* is the laser power, *f* is the repetition rate, *th* is the film thickness, α is the absorption coefficient, *σ_t_* is the pulse width, and *σ_xy_* is the spot size. The simulated transient photoacoustic pressure and acoustic streaming are shown in Figs. 2e and 2g respectively. The simulated temperature distribution at the film and water interface is shown in Fig. S4. The basic physical parameters of CNT-PDMS film including density, specific heat capacity, thermal conductivity, and thermal expansion coefficient were measured by Immersion method, Differential Scanning Calorimetry, Flash method, and Thermomechanical Analysis respectively and listed as below:

**Table S1. Material parameters of CNT-PDMS film**

| Material | Density  (kg/m^3^) | Specific heat capacity  (J/kg·K) | Thermal conductivity  W/(m·K) | Thermal expansion coefficient  (1/K) |
| --- | --- | --- | --- | --- |
| CNT-PDMS | 1036 | 1500 | 0.262 | 2.686×10^-4^ |

**Fig. S3.** Geometry of the simulation model.

**Fig. S4.** Temperature distribution at the interface between CNT-PDMS elastomer and water with different laser spot size. (a) Interface temperature with spot diameter of 200 µm. (b) Interface temperature with spot diameter of 300 µm.

**2.3. Surface Morphology Evolution of Elastomer Under Laser Illumination**

The surface morphology evolution of CNT-PDMS elastomer film was observed with microscope after 30 minutes photoacoustic laser streaming. Fig. S5 shows the surface morphologies of the film illuminated by laser with 200 μm and 300 μm spot diameters. The film illuminated by 200 μm laser exhibited deep dimple and obvious damage on the surface. In contrast, the surface of the sample driven by 300 μm laser shows only minor melt or deformation in local zone. Therefore, 300 μm laser was selected to performed high-speed PALS and corresponding liquid mixing applications.

**Fig. S5** CNT-PDMS elastomer film surface evolution after 30 minutes photoacoustic laser streaming with 200 μm and 300 μm laser spot diameters.

**2.4. Photoacoustic Streaming and Acoustic Signal Driven by Low Laser Power**

**Fig. S6.** Photoacoustic laser streaming (PALS) generated by laser with low laser power of 3 mW. (a) Snapshot of PALS of 3 mW laser driving. (b) Photoacoustic pressure under 3 mW laser illumination. The peak photoacoustic pressure is 0.22 MPa.

**2.5. Photoacoustic Streaming Time Evolution with Different Laser Repetition Rate**

The velocity measurement was conducted after a stabilization period of about 5 s to allow the fluid to accelerate from rest to its maximum stable velocity, and this procedure was applied to all measurements across different repetition rates and laser energies. As revealed by time-evolution flow imaging (Figs. S7–S9), the transient build-up phase typically requires only ~50 laser pulses (e.g., 500 ms for 100 Hz, and 250 ms for 200 Hz) for the fluid to accelerate from rest and form a stable photoacoustic jet. Therefore, this 5-second waiting period conservatively guarantees that all reported trajectory data were captured well within the steady-state regime.

**Fig. S7** Time evolution of the photoacoustic laser streaming with laser repetition rate of 100 Hz.

**Fig. S8** Time evolution of the photoacoustic laser streaming with laser repetition rate of 200 Hz.

**Fig. S9** Time evolution of the photoacoustic laser streaming with laser repetition rate of 300 Hz.

**2.6. Photoacoustic Laser Streaming with Different Laser Power and Repetition Rate**

**Fig. S10.** PALS generated with varying laser power at a fixed repetition rate of 100 Hz. (a-i) PALS generated by laser power of (a) 4 mW, (b) 5 mW, (c) 6 mW, (d) 7 mW, (e) 8 mW, (f) 9 mW, (g) 10 mW, (h) 15 mW, and (i) 20 mW respectively.

**Fig. S11** Fast Fourier Transform (FFT) spectrum of the ultrasonic signal with different laser power.

**Fig. S12.** PALS generated by laser with different repetition rates at a constant pulse energy around 0.22 mJ. (a-h) PALS generated by laser with repetition rate of (a) 100 Hz, (b) 200 Hz, (c) 300 Hz, (d) 400 Hz, (e) 500 Hz, (f) 1000 Hz, (g) 1500 Hz, and (h) 2000 Hz respectively.

Beyond the magnitude of the flow velocity, the spatial distribution (width and extension length) of the photoacoustic laser streaming is closely correlated with the total incident laser power. As observed in Figs. S7 and S9, whether the flow is driven by increasing the single-pulse energy or by elevating the pulse repetition rate, an increase in total laser power consistently leads to a higher flow velocity. With this increase in velocity, the fluid's forward momentum dominates, causing the jet to visually become narrower and highly directional.

The Reynolds number ($\text{Re=}\frac{\text{ρvL}}{\text{μ}}$) was calculated to confirm the viscosity-dominated regime and inertia-dominated regime in Fig. 3d and Fig. S12, where *ρ* is the density of water, *v* is the flow velocity, *L* is the characteristic length of the jet, and *μ* is the the dynamic viscosity. At low laser energy (0.03 mJ), the *Re* was calculated as *Re*=0.9 using velocity of 0.26 cm/s, *ρ*= 998.2 kg/m³, *μ*=1.002×10^-3^ Pa·s, and the characteristic length of the jet diameter *L*=350 µm. Because the *Re* <1, the flow is viscosity-dominated. At high laser energy, the *Re* was calculated as *Re*=78 using the velocity *v*=2.3 cm/s in Fig. 3c, the density of water *ρ*= 998.2 kg/m³, the dynamic viscosity *μ*=1.002×10^-3^ Pa·s, and estimating the characteristic length of the jet diameter *L*=3400 µm. Because *Re* ≫ 1, the flow is definitively governed by inertial forces rather than viscous drag in our high-speed regime*.* At the transition between the two regimes, the *Re* is about 17.6 for flow velocity of 1.47 cm/s (*L*=1200 μm) with laser power 6 mW and the *Re* is about 31.2 for flow velocity of 1.74 cm/s (*L*=1800 μm) with laser power 7 mW. The *Re* value shift (shifting from Re < 1 towards Re ~ 100) indicates the transition from inertial forces to viscous forces.

This high-speed, concentrated forward momentum effectively extends the driving range of the flow, allowing the jet to reach macroscopic extension lengths exceeding 4 cm (as demonstrated in Supplementary Video 2). Consequently, this long-range, highly directional spatial distribution is critical for penetrating stationary fluids, directly facilitating the rapid and efficient macroscopic liquid mixing demonstrated in our microfluidic applications.

**2.7. Photoacoustic Liquid Mixing**

**Fig. S13** Mixing process of blue ink with free diffusion. (a-d) Images of free-diffusion of blue ink in water at (a) 0 h, (b) 2 h, (c) 4 h, and (d) 12 h.

**Fig. S14** Photoacoustic micromixing with different flow rates. (a-c) Time-evolution snapshots of mixing at (a) 0 s, (b) 0.5 s, and (c) 1.5 s with flow rate of 1.2 µL/s. (d) Mixing efficiency of blue and red dyes in the microchannel with flow rate of 1.2 µL/s. (e-g) Time-evolution snapshots of mixing at (a) 0 s, (b) 3 s, and (c) 12 s with flow rate of 140 µL/s. (h) Mixing efficiency of blue and red dyes in the microchannel with flow rate of 140 µL/s.

**3. Video Caption**

Supporting video 1: High speed Photoacoustic laser streaming driven by laser with repetition rates of 2000 Hz and power of 440 mW.

Supporting video 2: Long-range photoacoustic laser streaming to around 4 cm.

Supporting video 3: Liquid mixing in cuvette with the CNT-PDMS photoacoustic transducer.

Supporting video 4: Laminar flow mixing in microchannel with photoacoustic CNT-PDMS micromixer.

**References**

1.	Tinevez, J.-Y.; Perry, N.; Schindelin, J.; Hoopes, G. M.; Reynolds, G. D.; Laplantine, E.; Bednarek, S. Y.; Shorte, S. L.; Eliceiri, K. W., TrackMate: An open and extensible platform for single-particle tracking. *Methods* **2017,** *115*, 80-90.

2.	Tropea, C., Yarin, A. L., & Foss, J. F. , *Springer handbook of experimental fluid mechanics*. Springer: Berlin, 2007; Vol. 1.

3.	Ahmed, D.; Mao, X.; Shi, J.; Juluri, B. K.; Huang, T. J., A millisecond micromixer via single-bubble-based acoustic streaming. *Lab Chip* **2009,** *9* (18).

4.	Bruus, H., Acoustofluidics 7: The acoustic radiation force on small particles. *Lab Chip* **2012,** *12* (6), 1014-21.

5.	Stokes, G. G., *On the Effect of the Internal Friction of Fluids on the Motion of Pendulums*. Cambridge University Press: 1850; p 38.

6.	Yue, S.; Lin, F.; Zhang, Q. H.; Epie, N.; Dong, S. C.; Shan, X. N.; Liu, D.; Chu, W. K.; Wang, Z. M.; Bao, J. M., Gold-implanted plasmonic quartz plate as a launch pad for laser-driven photoacoustic microfluidic pumps. *PNAS* **2019,** *116* (14), 6580-6585.

7.	Ai, X.; Lin, F.; Tong, T.; Chen, D.; Yue, S.; Mohebinia, M.; Napagoda, J.; Qiu, Y. A.; Tong, X.; Yu, P.; Chu, W. K.; Bao, J. M.; Wang, Z. M., Photoacoustic laser streaming with non-plasmonic metal ion implantation in transparent substrates. *Opt. Lett.* **2021,** *29* (14), 22567-22577.

8.	Wang, Y. N.; Zhang, Q. H.; Zhu, Z. A.; Lin, F.; Deng, J. D.; Ku, G.; Dong, S. C.; Song, S.; Alam, M. K.; Liu, D.; Wang, Z. M.; Bao, J. M., Laser streaming: Turning a laser beam into a flow of liquid. *Sci. Adv.* **2017,** *3* (9), e1700555.

9.	Zhang, T.; Ta, M.; Ke, D.; Ma, Y.; Wang, Z.; Gu, Z.; Huang, Y.; Yu, G.; Ouyang, J.; Yang, X.; Wei, W.; Guo, H.; Zhu, B., Precise regulation of photoacoustic frequency for all-optical ultrasound endoscopic imaging. *Photonics Res.* **2026,** *14* (3).
